# Association between anemia and frailty in 13,175 community-dwelling adults aged 50 years and older in China

**DOI:** 10.1186/s12877-019-1342-5

**Published:** 2019-12-04

**Authors:** Ye Ruan, Yanfei Guo, Paul Kowal, Ye Lu, Chazhen Liu, Shuangyuan Sun, Zhezhou Huang, Yang Zheng, Wenjing Wang, Gan Li, Yan Shi, Fan Wu

**Affiliations:** 1 Shanghai Municipal Centre for Disease Control and Prevention (Shanghai CDC), Shanghai, People’s Republic of China; 2 World Health Organization, Geneva, Switzerland; 3 Research Institute for Health Sciences, Chiang Mai University, Chiang Mai, Thailand; 4 School of public health, Shanghai Jiaotong university, Shanghai, People’s Republic of China; 5 Shanghai Medical College, Fudan University, Shanghai, People’s Republic of China

**Keywords:** Hemoglobin, Anemia, Frailty index, Ageing, China

## Abstract

**Background:**

Anemia and frailty contribute to poor health outcomes in older adults; however, most current research in lower income countries has concentrated on anemia or frailty alone rather than in combination. The aim of the present study was to investigate the association between anemia and frailty in community-dwelling adults aged 50 years and older in China.

**Methods:**

The study population was sourced from the 2007/10 SAGE China Wave 1. Anemia was defined as hemoglobin less than 13 g/dL for men and less than 12 g/dL for women. A Frailty Index (FI) was compiled to assess frailty. The association between anemia and frailty was evaluated using a 2-level hierarchical logistic model.

**Results:**

The prevalence of anemia was 31.0% (*95%CI*: 28.4, 33.8%) and frailty 14.7% (*95%CI*: 13.5, 16.0%). In the univariate regression model, presence of anemia was significantly associated with frailty (OR = 1.62, *95% CI*: 1.39, 1.90) and the effect remained consistent after adjusting for various potential confounding factors including age, gender, residence, education, household wealth, fruit and vegetable intake, tobacco use, alcohol comsumption and physical activity (adjusted OR = 1.31, *95% CI*:1.09, 1.57). Each 1 g/dL increase in hemoglobin concentration was associated with 4% decrease in the odds of frailty after adjusting for several confounding variables (adjusted OR = 0.96, *95% CI*: 0.93, 0.99).

**Conclusion:**

Anemia and low hemoglobin concentrations were significantly associated with frailty. Therefore, health care professionals caring for older adults should increase screening, assessment of causes and treatment of anemia as one method of avoiding, delaying or even reversing frailty.

## Background

China has a rapidly growing older adult population along with increasing life expectancy from 44.6 years in 1950 to 75.3 years in 2015, and is projected to reach almost 80 years by 2050 [1]. With ageing populations comes shifts in disease burdens, typically towards chronic non-communicable diseases. Low haemoglobin (Hb) in older adults increases the risk for a number of poor health outcomes, with anemia defined as hemoglobin less than 13 g/dL for men and less than 12 g/dL for women [2], increasing the levels of fatigue, cognitive decline and weakening muscle strength [3]. These same factors also contribute to frailty in older adults.

Frailty is a geriatric syndrome that increases vulnerability to stressors and leads to risk of negative outcomes such as falls, dependency, hospitalization and death [4]. While many tools were available to ascertain frailty, two tools were commonly used [5]. Rockwood defined frailty in terms of the accumulation of deficits (Frailty Index, FI), and generally including 30–40 variables [6]. Fried suggested a frailty phenotype identified by the presence of three or more of five components (unintentional weight loss, weakness, poor endurance and energy, slowness and low physical activity) [7]. The prevalence of frailty varied from 4 to 59.1% with different assessment and geographic region [8].

The burden imposed by the co-occurrence of anemia and frailty in older age posed a potential challenge for healthcare systems worldwide. A recent meta-analysis estimated that older persons with anemia had more than double the odds of frailty, although with conflicting results for the two longitudinal studies (2–5 years follow-up) that assessed the association between anemia and frailty [9]. Many of the studies that contributed to these estimates were focused on high-income countries, and used the phenotype criteria [7] to define frailty. To our knowledge, there have been no large representative studies to assess the relationship between anemia and frailty in older community-dwelling population in China.

The aim of the present study was to investigate the association between anemia and frailty in community-dwelling adults aged 50 years and older from the World Health Organization Study on global AGEing and adult health (SAGE) China Wave 1.

## Methods

### Study population and design

SAGE was a longitudinal cohort study of ageing and older adults in six low- and middle-income countries (China, Ghana, India, Mexico, Russian Federation and South Africa) [10]. The study population was sourced from SAGE China Wave 1 from 2007 to 2010, using a probability sampling design and a five-stage cluster sampling strategy [11]. SAGE China Wave 1 contacted 1642 individual respondents aged 18–49 years and 13,367 respondents aged 50+ years. The response rate for the individual questionnaire was 98%, and a final total sample size of 13,175 for this analysis.

SAGE was approved by the World Health Organization’s Ethical Review Board (RPC146), and local approval by the ethics review committee of the Chinese Center for Disease Control and Prevention (approval notice 200,601). Each respondent signed informed consent.

### Measures

#### Anemia

Blood hemoglobin concentrations were derived from dry blood spot samples and examined using standardized enzyme-linked immunosorbent assay (ELISA) techniques at the laboratory in Shanghai Municipal Centre for Disease Control and Prevention. The World Health Organization’s (WHO) definition of anemia was used: Hb less than 13 g/dL for men and less than 12 g/dL for women [2].

#### Frailty

Frailty was defined using the deficit accumulation approach. A Frailty Index (FI) was generated as the proportion of deficits present out of 40 variables available in the SAGE database, including self-rated health, 9 medically diagnosed conditions, 4 medical symptoms,13 functional activities assessments, 10 activities of daily living (ADLs), body mass index (BMI, calculated as weight/height^2^(kg/m^2^)), grip strength and gait speed [12]. Individual scores ranged from 0 (no deficits) to 1 (highest level of deficits in all variables). The FI cut-off value of 0.2 was defined as approaching a frail state [12].

#### Other covariates

SAGE used a standardized survey instrument to collect sociodemographic information and behavioral risk factors based on the WHO STEPwise approach to Surveillance (WHO STEPS, WHO 2005). Socio-demographic variables included age, sex, education, rural/urban residence, and household wealth. All respondents were categorized into four age groups: 50–59, 60–69,70–79 and 80 years or older. The international classification scheme was used to classify the level of education into six categories (No formal education, less than primary, primary school completed, secondary school completed, high school completed, college completed and above) [13]. The household wealth quintiles were generated using an asset-based approach, ranging from quintile1 (Q1, lowest) to quintile 5 (Q5, highest) [14]. Alcohol and tobacco consumption, low physical activity level and insufficient fruit and vegetable intake were included as common non-communicable disease risk factors. The individuals were classified into four groups (never smoker, not current smokers, current smokers (not daily) and current daily smokers) on the basis of the form and amount of tobacco use. Alcohol consumption had four groups: never drinker, non-heavy drinkers, infrequent heavy drinkers and frequent heavy drinkers by calculating the number of alcoholic drinks consumed within a given week. The Global Physical Activity Questionnaire (GPAQ) was used to generate three categories of physical activity: low, moderate and high levels [15]. Insufficient fruit and vegetable intake was defined as eating fruit and vegetable less than five servings (equivalent to at least 400 g per day) [16].

### Statistical methods

Statistic analyses were conducted using STATA SE version 14.1 (Stata Corp, College Station, TX). The population prevalence of anemia and frailty was calculated by using normalized weights. Weights were based on selection probability, non-response, and post-stratification adjustments. A 2-level hierarchical logistic model was used to evaluate the association between anemia and frailty using STATA command “melogit”. We also included hemoglobin concentration as a continuous variable in the model 3 and 4 to see if there was an association between hemoglobin concentration and frailty. Covariates of interest included age, gender, residence, education, household wealth, fruit and vegetable intake, tobacco use, alcohol comsumption and physical activity. *P* < 0.05 from two-sided statistical tests was considered statistically significant.

## Results

The sociodemographic characteristics of samples were shown in Table 1. A total of 13,175 individuals aged 50 and older were included in the analysis. The proportion of women (50.2%) was higher than men (49.8%) in the study, with small sex differences by age groups. The overall mean age was 62.6 years (SE 0.2). The majority of the respondents were between 50 and 59 years old (44.9%), nearly half of all respondents (47.3%) lived in an urban area. Fifty-eight percent had completed primary school or higher. The prevalences of lowest and highest wealth quintile were 16.3 and 21.8% respectively.

**Table 1:** Sociodemographic characteristics of the study population, by sex, SAGE China Wave 1

	Men(*n* = 6171)				Women(*n* = 7004)				Total(*n* = 13,175)			
	n	%	N	%	n	%	N	%	n	%	N	%
			(Weighted)	(Weighted)			(Weighted)	(Weighted)			(Weighted)	(Weighted)
Mean age (SE)	63.2	−0.1	62.1	−0.2	63.1	−0.1	63	−0.2	63.2	−0.1	62.6	−0.2
Age												
50–59	2636	42.7	3061	46.8	3065	43.8	2851	43.1	5701	43.3	5912	44.9
60–69	1868	30.3	2132	32.6	2058	29.4	2061	31.2	3926	29.8	4192	31.9
70–79	1319	21.4	1091	16.7	1453	20.7	1356	20.5	2772	21	2447	18.6
80+	348	5.6	262	4	428	6.1	343	5.2	776	5.9	606	4.6
Residence												
Urban	2846	46.1	2876	43.9	3582	51.1	3353	50.7	6428	48.8	6229	47.3
Rural	3325	53.9	3670	56.1	3422	48.9	3258	49.3	6747	51.2	6928	52.7
Education												
No formal education	898	14.6	858	13.1	2451	35	2178	32.9	3349	25.4	3036	23.1
Less than primary	1107	17.9	1207	18.4	1241	17.7	1285	19.4	2348	17.8	2492	18.9
Primary school completed	1435	23.3	1602	24.5	1157	16.5	1163	17.6	2592	19.7	2766	21.0
Secondary school completed	1414	22.9	1528	23.3	1195	17.1	1084	16.4	2609	19.8	2612	19.9
High school completed	916	14.8	952	14.6	764	10.9	708	10.7	1680	12.8	1660	12.6
College completed and above	401	6.5	399	6.1	196	2.8	193	2.9	597	4.5	592	4.5
Wealth Quintile												
Q1(Lowest)	1186	19.3	1032	15.8	1442	20.7	1099	16.7	2628	20	2131	16.3
Q2	1239	20.1	1198	18.4	1365	19.6	1176	17.9	2604	19.9	2374	18.1
Q3	1268	20.6	1342	20.6	1373	19.7	1341	20.4	2641	20.1	2684	20.5
Q4	1271	20.7	1530	23.5	1412	20.3	1529	23.2	2683	20.5	3059	23.4
Q5(Highest)	1186	19.3	1417	21.7	1372	19.7	1432	21.8	2558	19.5	2849	21.8

The mean Hb level was 13.3 ± 3.0 g/dL, being 14.0 ± 3.0 g/dL in men and 12.8 ± 2.8 g/dL in women respectively (2633 of 13,175 blood samples were missing). Overall prevalence of anemia was 31.0% (*95%CI*: 28.4, 33.8%)(Table 2). By gender, 31.7% of men and 30.3% of women were found to be anemic (F = 3.103, *P* = 0.048). The prevalence of anemia among rural dwelling respondents (19.4%) was lower than in urban areas (46.3%)(F = 76.318, *P* < 0.001). Anemia prevalence was higher in older age groups. Higher wealth individuals had higher anemia rates, reaching 39.7% (*95%CI*: 34.1, 45.5%) in the richest group. In contrast, the prevalence of anemia decreased (F = 4.656, P < 0.001) at higher levels of education.

**Table 2:** Prevalence of anemia^a^ among adults aged 50 years and older, SAGE China Wave 1

	Men (*n* = 5044)		Women (*n* = 5482)		Total (*n* = 10,526)	
	%	*95%CI*	%	*95%CI*	%	*95%CI*
**Total**	31.7	[29.0, 34.5]	30.3	[27.3, 33.5]	31.0	[28.4, 33.8]
Age						
50–59	26.5	[22.7, 30.7]	29.4	[26.2, 32.8]	27.9	[24.7, 31.2]
60–69	30.2	[26.2, 34.4]	29.8	[26.2, 33.7]	30.0	[26.8, 33.5]
70–79	38.5	[33.4, 43.8]	37.8	[33.2, 42.5]	38.1	[33.7, 42.7]
80+	43.8	[36.2, 51.7]	38.9	[31.1, 47.4]	41.2	[35.5, 47.1]
F	9.628		3.231		4.221	
P	< 0.001		0.006		0.002	
Residence						
Urban	48.5	[42.6, 54.5]	44.3	[39.9, 48.8]	46.3	[41.5, 51.2]
Rural	18.3	[15.7, 21.2]	20.7	[17.6, 24.0]	19.4	[16.8, 22.2]
F	94.443		59.367		76.318	
P	< 0.001		< 0.001		< 0.001	
Education						
No formal education	33.0	[28.0, 38.4]	35.4	[31.6, 39.5]	34.7	[31.2, 38.5]
Less than primary	27.1	[22.3, 32.6]	29.9	[25.8, 34.3]	28.5	[24.6, 32.7]
Primary school completed	29.8	[24.8, 35.3]	33.7	[29.6, 38.2]	31.4	[27.3, 35.7]
Secondary school completed	32.9	[27.9, 38.4]	28.5	[23.6, 33.9]	31.2	[26.8, 35.9]
High school completed	29.4	[20.5, 40.4]	27.5	[21.2, 34.8]	28.6	[21.5, 37.1]
College completed and above	29.5	[17.8, 44.8]	20.0	[12.2, 31.0]	26.1	[16.2, 39.1]
F	0.515		3.862		4.656	
P	0.653		< 0.001		< 0.001	
Wealth Quintile						
Q1(Lowest)	26.0	[21.9, 30.6]	33.5	[28.8, 38.6]	29.8	[26.0, 33.9]
Q2	29.0	[25.1, 33.3]	31.0	[26.3, 36.1]	30.0	[26.1, 34.2]
Q3	29.0	[24.7, 33.6]	30.4	[26.6, 34.5]	29.7	[26.2, 33.4]
Q4	25.9	[21.2, 31.2]	28.2	[24.4, 32.2]	27.0	[23.1, 31.2]
Q5(Highest)	42.3	[35.3, 49.7]	36.9	[32.1, 42.0]	39.7	[34.1, 45.5]
F	8.557		4.624		7.400	
P	< 0.001		< 0.001		< 0.001	

^a^Weighted results

Frailty prevalence was 14.7% (*95%CI*: 13.5, 16.0%), being higher in women (17.4%) than men (11.9%)(F = 52.933, P < 0.001) (Table 3). The 80+ age group had the highest prevalence of frailty (41.2%). Compared with urban respondents, rural dwellers had higher levels of frailty (15.6%). Lower education and wealth levels were associated with higher frailty (P < 0.001).

**Table 3:** Prevalence of frailty^a^ among adults aged 50 years and older, SAGE China Wave 1

	Men (*n* = 6124)		Women (*n* = 6949)		Total (n = 13,070)	
	%	*95%CI*	%	*95%CI*	%	*95%CI*
Total	11.9	[10.6, 13.3]	17.4	[15.9, 19.1]	14.7	[13.5, 16.0]
Age						
50–59	5.7	[4.6, 7.1]	10.0	[8.5, 11.8]	7.8	[6.7, 9.0]
60–69	12.9	[11.1, 14.9]	17.2	[14.9, 19.8]	15.0	[13.4, 16.8]
70–79	21.3	[18.3, 24.6]	27.1	[23.9, 30.6]	24.5	[22.1, 27.0]
80+	38.0	[31.5, 45.1]	43.6	[36.9, 50.6]	41.2	[36.3, 46.3]
F	84.699		65.848		135.252	
P	< 0.001		< 0.001		< 0.001	
Residence						
Urban	11.4	[9.3, 13.8]	15.6	[13.5, 18.0]	13.7	[11.8, 15.8]
Rural	12.3	[10.8, 14.0]	19.3	[17.2, 21.6]	15.6	[14.2, 17.2]
F	0.487		5.272		2.281	
P	0.487		0.024		0.134	
Education						
No formal education	19.1	[16.0, 22.7]	25.4	[23.1, 28.0]	23.6	[21.6, 25.9]
Less than primary	12.4	[10.1, 15.0]	17.4	[14.2, 21.1]	15.0	[12.8, 17.4]
Primary school completed	13.2	[11.0, 15.8]	14.1	[11.7, 17.0]	13.6	[11.7, 15.8]
Secondary school completed	8.9	[6.9, 11.4]	11.2	[8.8, 14.1]	9.8	[8.2, 11.8]
High school completed	8.8	[6.7, 11.5]	10.4	[7.7, 13.8]	9.5	[7.7, 11.6]
College completed and above	8.8	[5.5, 13.7]	8.3	[4.7, 14.2]	8.6	[5.5, 13.3]
F	8.183		19.881		29.388	
P	< 0.001		< 0.001		< 0.001	
Wealth Quintile						
Q1(Lowest)	17.8	[15.1, 20.9]	23.0	[20.2, 26.1]	20.5	[18.3, 22.9]
Q2	15.5	[12.8, 18.7]	21.1	[18.1, 24.3]	18.3	[16.1, 20.7]
Q3	12.4	[10.5, 14.7]	19.5	[17.1, 22.3]	16.0	[14.3, 17.8]
Q4	10.0	[8.1, 12.2]	14.5	[12.1, 17.3]	12.2	[10.4, 14.3]
Q5(Highest)	5.5	[3.7, 8.0]	10.3	[7.8, 13.5]	7.9	[6.1, 10.2]
F	16.200		14.101		24.866	
P	< 0.001		< 0.001		< 0.001	

^a^Weighted results

Table 4 shows the associations between anemia and frailty for all respondents. In the univariate regression model (model 1), presence of anemia was significantly associated with frailty (OR = 1.62, *95% CI*: 1.39, 1.90) and the effect attenuated only slightly after adjusting for various potential confounding factors including age, gender, residence, education, household wealth, fruit and vegetable intake, tobacco use, alcohol comsumption and physical activity (model 2) (adjusted OR = 1.31, *95% CI*:1.09, 1.57). Further, we included hemoglobin concentration in the model 3 and 4 to examine the associations, and found each 1 g/dL increase in hemoglobin concentration was associated with 4% decrease in the odds of frailty after adjusting for age, gender, residence, education, household wealth, fruit and vegetable intake, tobacco use, alcohol comsumption and physical activity (adjusted OR = 0.96, *95% CI*: 0.93, 0.99).

**Table 4:** Odds ratios (95% confidence intervals) for frailty, unadjusted and adjusted by covariates

Variables	Model1	Model2	Model3	Model4
	OR (*95%CI*)	Adjusted OR (*95%CI*)	OR (*95%CI*)	Adjusted OR (*95%CI*)
Anemia	1.62**[1.39, 1.90]	1.31**[1.09, 1.57]	\	\
Hb concentration	\	\	0.90**[0.87, 0.92]	0.96**[0.93,0.99]
Age				
50–59		ref		ref
60–69		1.99** [1.69, 2.35]		1.98** [1.68, 2.34]
70–79		3.22** [2.66, 3.89]		3.19** [2.64, 3.86]
80+		7.14** [5.45, 9.35]		7.12** [5.43, 9.33]
Gender				
Men		ref		ref
Women		1.23* [1.03, 1.46]		1.18 [0.99, 1.41]
Residence				
Urban		ref		ref
Rural		0.92 [0.62, 1.37]		0.93 [0.62, 1.37]
Education				
No formal education		ref		ref
Less than primary		0.75** [0.62, 0.91]		0.75**[0.62, 0.92]
Primary school completed		0.68** [0.56, 0.83]		0.69**[0.56, 0.84]
Secondary school completed		0.71** [0.56, 0.90]		0.72**[0.57, 0.91]
High school completed		0.66** [0.50, 0.87]		0.66**[0.50, 0.88]
College completed and above		0.59* [0.39, 0.89]		0.60*[0.40, 0.91]
Wealth quintile				
Q1(Lowest)		ref		ref
Q2		0.87 [0.72, 1.06]		0.87 [0.72, 1.05]
Q3		0.72** [0.59, 0.89]		0.72**[0.58, 0.89]
Q4		0.69** [0.55, 0.86]		0.68**[0.58, 0.89]
Q5(Highest)		0.59** [0.44, 0.78]		0.58**[0.44, 0.77]
Fruit and vegetable intake				
Insufficient		ref		ref
Sufficient		0.66** [0.57, 0.77]		0.66**[0.57, 0.76]
Alcohol consumption				
Never drinker		ref		ref
Non-heavy drinkers		0.78* [0.63, 0.97]		0.79*[0.63, 0.97]
Infrequent heavy drinkers		1.36 [0.72, 2.59]		1.36 [0.71, 2.59]
Frequent heavy drinkers		0.75 [0.51, 1.09]		0.76 [0.52, 1.10]
Tobacco use				
Never smoker		ref		ref
Not current smoke		1.12 [0.80, 1.56]		1.11 [0.80, 1.56]
Smoker, not daily		0.97 [0.59, 1.60]		0.98 [0.59, 1.62]
Current daily smoker		0.93 [0.76, 1.13]		0.93 [0.76, 1.14]
Physical activity				
Lower level		ref		ref
Moderate level		0.54** [0.46, 0.64]		0.54**[0.46, 0.63]
High level		0.36** [0.30, 0.42]		0.36**[0.30, 0.42]
Constant		0.23** [0.15, 0.36]		0.46*[0.25, 0.85]

*P < 0.05, **P < 0.01

## Discussion

This study reported the prevalences of anemia and frailty and the two conditions combined in a large population of older Chinese adults. The prevalences of both conditions were higher at older ages and in individuals with lower education levels. In addition, anemia was significantly associated with frailty, where each 1 g/dL increase in hemoglobin concentration was related with 4% decrease in the odds of frailty after adjusting for several variables. As far as we know, this was the first paper addressing the association between anemia and frailty among community-dwelling adults aged 50 years and older in China.

While estimates of anemia prevalence differed considerably, with reported prevalence ranging from 2.9 to 61% in older men and from 3.3 to 41% in older women [17], the prevalence was generally higher in men than in women and increased with advancing age [17, 18]. Anemia prevalence was 14.1% for men and 10.2% for women aged 65 and older in the US National Health and Nutrition Examination Survey (NHANES 2013–2016) [19]. An Australian epidemiologic study had anemia estimates of 14.6% among men aged 70+ years [20]. Thirty-eight percent of community-dwelling people aged 60 years and older had anemia in a small study in India [21]. Likewise, 38.1% of older adults had anemia in the Singapore Longitudinal Ageing Studies (SLAS) [22]. Our analyses indicated that the prevalence of anemia was 31.0% (95%CI: 28.4–33.8%) in China, which was higher in men, older people, lower levels of education, and those lived in urban area and with higher wealth, being inverse of some results among older Mexican adults [23]. Considering hemoglobin is an indicator for malnutrition in older population, the contrast may be due to the different distribution of missing data among income groups in our research. If those with missing data disproportionately fell into the higher/lower income group, the prevalence of anemia will be underestimated/overestimated. In addition, the different population, sampling programs and hemoglobin test methods may also contribute to the difference between these studies.

Attention to the measurement and impact of frailty in older age has increased substantially over the past decade. For example, the overall weighted prevalence of frailty was 9.9% in the community-dwelling older population (60+ years) derived from the China Comprehensive Geriatric Assessment Study (CCGAS), based on the Comprehensive Geriatric Assessment Frailty Index [24]. The physical frailty phenotype approach was used in an analysis of the China Health and Retirement Longitudinal Study (CHARLS), resulting in 7% of adults aged 60 years or older being classified as frail [25]. In our research, 14.7% (95%CI: 13.5–16.0%) of community-dwelling residents aged 50^+^ years were frail by using FI assessment, higher than the two studies mentioned which use somewhat different frailty criteria.

Anemia reduces the oxygen-carrying capacity, which can result in tissue hypoxia and lead to a number of poor outcomes, including reduced submaximal and maximal aerobic capacity, failing muscle strength, cognitive impairment and development of frailty [26–28], which related to vulnerability and some negative outcomes. Several previous studies have examined the interaction between anemia and frailty among older people in high income countries. A case-control study in Baltimore (USA) firstly explored the relationship between anemia and frailty, showing an inverse correlation between interleukin-6 (IL-6) and hemoglobin or hematocrit in the frail group, suggesting that frail subjects have evidence of inflammation and lower hemoglobin and Hematocrit levels [29]. Data from the Women’s Health and Aging Studies (WHAS) I and II found that mildly low and low-normal hemoglobin levels were associated with increased frailty, and the risk of frailty increased at statistically significant levels for anemia adjusted for age, race, and education [30, 31]. Another cross-sectional and longitudinal study in older Australian men also suggested that anemia may contribute to the development of frailty [20]. Recent studies including both older men and women indicated that older anemic adults were more likely to be frail, with the association between lower levels of hemoglobin and number of frailty criteria showing dose-response effect [32–34]. However, another contrasting result suggested having anemia contributed to a weak but significantly lower chance of worsening frailty [35]. In our study, we used 40 variables to construct a Frailty Index and observed that both anemia and lower concentrations of hemoglobin were associated with frailty.

Some studies have suggested that age-associated chronic inflammation is an explanatory factor in the relationship between anemia and frailty. In older adults, anemia and frailty may share a pathophysiological pathway with chronic inflammatory processes, resulting from immunosenescence-associated changes and increased oxidative stress [36–38]. Gabriele [39] described a close connection between inflammaging, anemia, and frailty, where comorbidities and inflammaging contribute to anemia of chronic inflammation (ACI), which was the most frequent type of anemia in older adults. Considering the etiopathogenetic mechanisms of inflammation, some interventions such as dietetic approach and physical exercise that can moderate oxidative stress and chronic inflammation may prevent anemia, frailty and their negative impact on functional performance and quality of life. Another study reported that a high intake of dietary total antioxidant capacity (TAC) was inversely related with frailty, and the intake of green tea, vegetable and fruits which contributed to TAC was also associated with lower odds of frailty [40]. Our results also indicated sufficient intake of vegetables and fruit and moderate to high levels of physical exercise had protective effects against frailty.

There were a few limitations in our study. Firstly, we used cross-sectional data from SAGE China Wave 1, it cannot provide causal direction in the relationship between anemia and frailty. Results from SAGE China Waves 2 and 3 may provide an opportunity to examine the direction of this relationship we identified. Secondly, we used self-report for some items to construct Frailty Index, which may be influenced by recall bias, although self-reported health questions were widely applied in population studies. Thirdly, the missing data for haemoglobin may have also contributed to selection bias. We analyzed the distribution of the missing data of Hb and found that total of missing values were randomly distributed across five income groups, but there were significant differences between rural and urban across the income groups, that might be the reason why higher wealth individuals had higher anemia rates. However, our study was based on a large, national probability and representative sample of older adults of both genders in China. Furthermore, the results indicated a quantitative relationship between hemoglobin concentration and frailty.

## Conclusions

In conclusion, anemia and frailty were prevalent in China dwelling adults aged 50 years and older, and we also found that anemia and lower levels of hemoglobin concentration were significantly associated with frailty. Therefore, health care professionals caring for older adults may want to improve their recognition and treatment of anemia in their patient populations. Attention at the primary care level may reduce this risk for frailty, disability, hospitalization and mortality. This way, effective policies, early screening and health interventions can be employed for avoiding, delaying or even reversing frailty in a rapidly growing population in China.

## Data Availability

The datasets supporting the conclusions of this article are available upon request in the website of WHO (http://apps.who.int/healthinfo/systems/surveydata/index.php/catalog/sage).

## Abbreviations

ACI: Anemia of chronic inflammation
ADLs: Activities of daily living
BMI: Body mass index
CCGAS: China Comprehensive Geriatric Assessment Study
CHARLS: China Health and Retirement Longitudinal Study
ELISA: Enzyme-linked immunosorbent assay
FI: Frailty Index
GPAQ: Global Physical Activity Questionnaire
Hb: Haemoglobin
IL-6: Interleukin-6
NHANES: National Health and Nutrition Examination Survey
SAGE: Study on global AGEing and adult health
SLAS: Singapore Longitudinal Ageing Studies
TAC: Total antioxidant capacity
WHAS: Women’s Health and Aging Studies
WHO: World Health Organization
